# Drug-induced interstitial lung disease associated with dupilumab for the treatment of atopic dermatitis

**DOI:** 10.1016/j.jdcr.2024.05.018

**Published:** 2024-05-20

**Authors:** Bethany F. Wilken, Onofre Moran-Mendoza, Marina Pourafkari, Yuka Asai

**Affiliations:** a Division of Dermatology, Department of Medicine, Queen’s University, Kingston, Ontario, Canada; b Division of Respirology and Sleep Medicine, Department of Medicine, Queen’s University, Kingston, Ontario, Canada; c Division of Diagnostic Radiology, Department of Medicine, Queen’s University, Kingston, Ontario, Canada

**Keywords:** adverse event, atopic dermatitis, drug-induced interstitial lung disease, dupilumab

## Introduction

Atopic dermatitis (AD) is a chronic condition marked by T-helper (Th)2-mediated inflammation and excess interleukin (IL)-4 and IL-13 production. Dupilumab, a monoclonal antibody inhibiting IL-4 and IL-13 signaling, is used in AD and has approval for various other conditions. Common adverse effects include conjunctivitis, blepharitis, injection site reactions, and, more recently noted, Th17-predominant diseases.^1^ Here, we report a case of interstitial lung disease (ILD) developing after dupilumab treatment for severe AD.

## Case reports

A 73-year-old nonsmoking woman with long-standing severe AD presented to the dermatology clinic on a short course of oral prednisone to treat an AD flare. She was started on dupilumab after the failure of topical therapies and methotrexate. The patient cleared on dupilumab with Investigator Global Assessment and Eczema Area and Severity Index (EASI) scores of 0 at 12, 24, and 36 months.

At 28 months into dupilumab treatment, the patient complained of a persistent dry cough, reportedly progressing over 2 years. She had stopped methotrexate and prednisone 27 months prior and did not have fever, chills, myalgia, weight loss, or night sweats. Her white blood cell count was 7 × 10^9^/L (normal: 3.5-10.5 × 10^9^/L), and eosinophils were 0.1 × 10^9^/L (normal: 0-0.5 × 10^9^/L). Postnasal drip and rhinosinusitis were ruled out by an otorhinolaryngologist. She was referred to an allergy, and the cough was believed to be a variant of uncontrolled asthma. She was given montelukast 10 mg p.o. and budesonide/formoterol 60/4.5 mcg 2 inhalations twice a day, which were ineffective.

**Fig 1:**
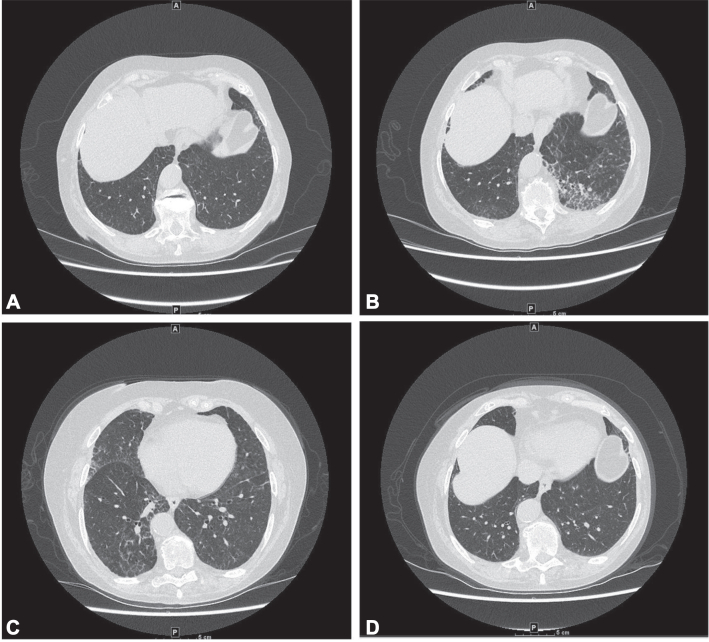
**A,** Chest high-resolution CT imaging shortly after the initial visit to ILD clinic showed mild reticulations in right lower lobe. **B,** Worsening interstitial changes with reticulation, mostly in the left lower lobe, despite removal of foam pillows. **C,** Improvements in the ground glass appearance and reticulations in left lower lobe, yet progressive changes in the right middle lobe 2 months after dupilumab cessation. **D,** Improvements in reticulations in the right middle lobe, right lower lobe, and left lower lobe 10 months after dupilumab cessation. *CT*, Computed tomography; *ILD*, interstitial lung disease.

Due to the persistent cough, the patient had a high-resolution chest computed tomography (HRCT) that showed interstitial changes, and she was referred to the ILD clinic. The HRCT revealed reticulations in the right lower lobe, consistent with probable usual interstitial pulmonary fibrosis (Fig 1, *A*). Bronchoalveolar lavage (BAL) fluid showed normal eosinophil levels but significant lymphocytosis (84%; normal 5% to 15%) with mild neutrophilia (10%; normal ≤3%), suggesting hypersensitivity pneumonitis (HP). No skin signs of connective tissue disease (CTD) were observed, such as periungual capillaritis or other dermatitis, and serologic investigations for CTD were negative including rheumatoid factor, antinuclear, double-stranded DNA, extractable nuclear antigen, anti-neutrophil cytoplasmic and cyclic citrullinated peptide antibodies, and creatine kinase, which was normal. Interestingly, analysis of the patient’s foam pillows by an environmental microbiology lab uncovered the presence of molds that have been associated with HP (*Alternaria and Penicillium sp*.).

Despite switching to polyester pillows and encasing the pillows and mattress, the cough persisted, now accompanied by dyspnea and clear sputum. A follow-up of HRCT 4 months later indicated worsening interstitial changes with reticulation (Fig 1, *B*). The patient's medications and their timelines were reviewed (dupilumab, amlodipine, lansoprazole, montelukast, magnesium citrate, calcium, vitamin D3, and vitamin B12) in consideration of a diagnosis of drug-induced interstitial lung disease (DILD). The patient declined a lung biopsy and chose to discontinue dupilumab.

**Fig 2:**
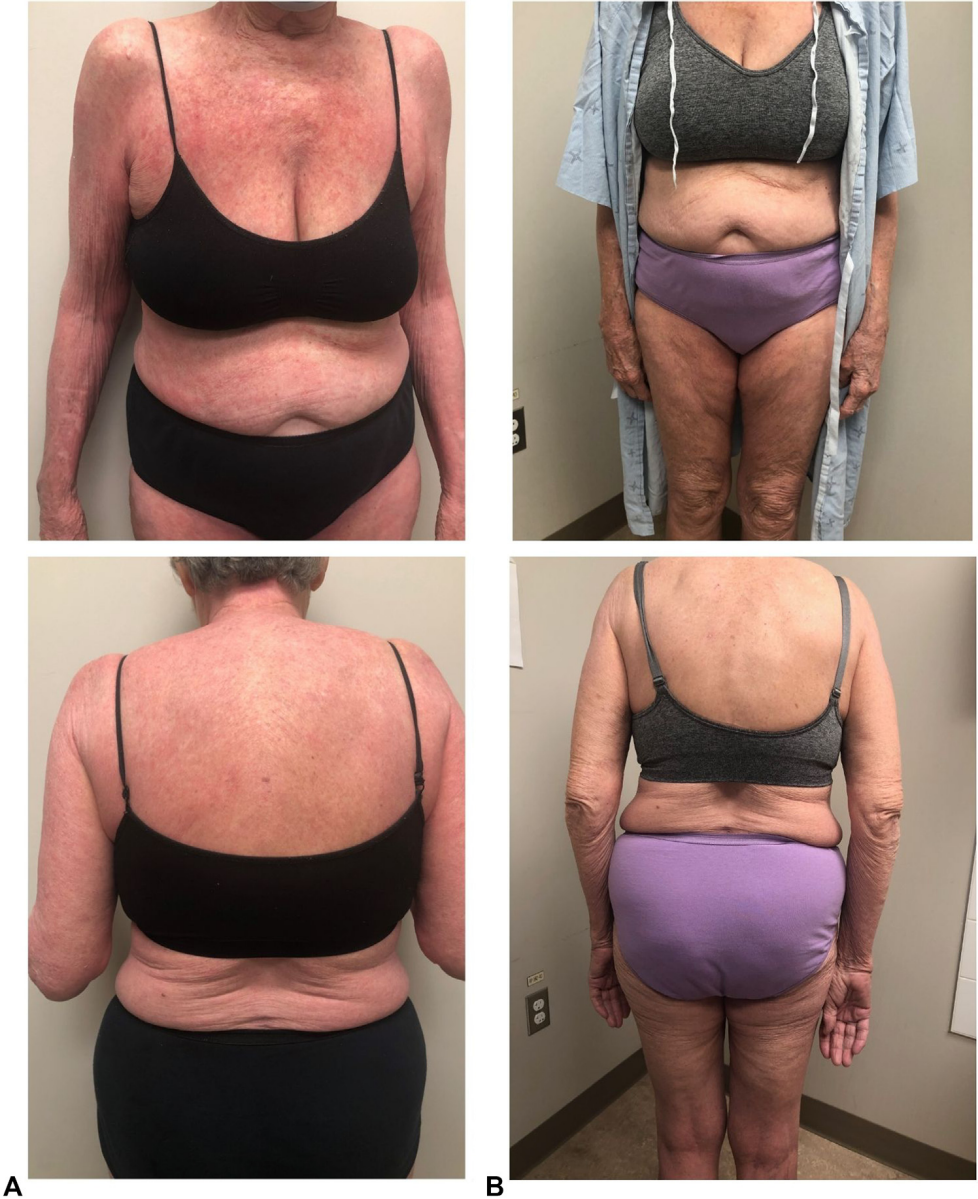
**A,** Patient images taken 6 weeks after dupilumab cessation showed severe AD with erythroderma. Body surface area (BSA): 97%, EASI score: 61. **B,** AD is clear 3 months after starting tralokinumab. EASI score: 0. *AD*, Atopic dermatitis; *EASI*, Eczema Area and Severity Index.

Six weeks after dupilumab cessation, the patient presented to the dermatology clinic with erythroderma (EASI 61) (Fig 2, *A*). She was given 30 mg of oral prednisone for a week with a taper of 5 mg every 3 days, and a request for tralokinumab was submitted. At 3 months on tralokinumab, the patient was clear (EASI 0) (Fig 2, *B*).

Two months post-dupilumab cessation, the patient reported no improvement in her cough, and the HRCT showed mixed results with resolution of some previous lung changes but progressive interstitial changes of the right middle lobe (Fig 1, *C*). Six months after discontinuation, the patient reported that her chronic cough had significantly improved by 95%. At 10 months post-dupilumab cessation, there was normal lung function, complete resolution of ILD, and progressive improvements in dyspnea, cough, pulmonary function, and imaging. Reticulations and the probable usual interstitial pulmonary fibrosis pattern observed in the initial HRCT were undetectable (Fig1, *D*).

## Discussion

A diagnosis of DILD can be difficult to make, but the key criteria supporting a diagnosis are a clear temporal association between the causative agent and the development/resolution of respiratory symptoms, pulmonary function test abnormalities, and radiologic findings.^2^ These criteria were met, and other causes of the patient’s ILD, including CTD, infection, and malignancy, were excluded through clinical assessments, serology, and BAL. Review of product monographs and consultation of the Pneumotox website (https://www.pneumotox.com/drug/index/) showed no reported association with DILD and the patient's other medications. The Naranjo Scale is used to determine the likelihood that a particular medication caused an adverse reaction in a patient. Based on 10 questions, the total score helps to categorize the probability of the medication causing the adverse reaction, ranging from doubtful (score ≤ 0) to definite association (≥9).^3^ The patient scored 7 on the Naranjo scale, indicating a probable adverse drug reaction to dupilumab.

Symptoms and radiographic abnormalities of DILD caused by biologic therapies generally develop weeks to months after treatment initiation and improve 2 to 6 months after drug discontinuation.^4^ This aligns with the patient's CT improvements 2 months after dupilumab cessation and the resolution of her cough at 6 months. Resolution of symptoms in foam-related HP can occur almost immediately^5^; if the pillows were the cause, improvement or cessation of her symptoms would be expected at follow-up. Instead, the symptoms and CT scans worsened before improving after dupilumab discontinuation. While we cannot state with absolute certainty without a lung biopsy, which the patient declined, these features are strongly suggestive of DILD from dupilumab, classified as Grade 3 (severe) due to the severity of symptoms and impact on daily life.^6^

Dupilumab-related pulmonary adverse events have been reported, such as hypereosinophilia and chronic eosinophilic pneumonia in asthma treatment.^7^^,^^8^ Notably, our patient exhibited no elevation in blood eosinophils or criteria for eosinophilic pneumonia in BAL. A mechanistic explanation for dupilumab-induced DILD is unknown, but studies suggest that IL-4 suppression leads to increased Th17 cell activity, producing pro-inflammatory cytokines linked to pulmonary inflammation and fibrosis.^9^

As dupilumab indications are expanding for other allergic/inflammatory conditions and as the symptoms of DILD may initially present like those of other atopic diseases, it is our aim to raise awareness among clinicians and researchers, facilitating the identification of other cases of dupilumab-related DILD and the underlying pathophysiological mechanisms.

## Conflicts of interest

Dr Asai declares honoraria and speaker fees from Sanofi, Pfizer, AbbVie, Leo, Sun Pharma, Novartis, Miravo, Eli Lilly, Kyowa Kirin, and L’Oreal; research and education grants from Sanofi Canada, AbbVie, Pfizer, Novartis, the Canadian Dermatology Foundation, and the Eczema Society of Canada; and performs clinical trials for Novartis and Leo. Author Wilken and Drs Moran-Mendoza and Pourafkari have no conflicts of interest to declare.
